# Phosphorylated mTOR Expression Profiles in Human Normal and Carcinoma Tissues

**DOI:** 10.1155/2017/1397063

**Published:** 2017-07-31

**Authors:** Hojung Lee

**Affiliations:** Department of Pathology, Nowon Eulji Medical Center, Eulji University, Seoul, Republic of Korea

## Abstract

Mammalian target of rapamycin (mTOR) is a key controller of cell growth and proliferation in normal tissues and solid tumors. In the present study, an immunohistochemical analysis of the expression pattern of phosphorylated mTOR (p-mTOR) was performed in human normal fetal and adult tissues and various carcinoma tissues. p-mTOR expression showed tissue and cell type specificity in normal and cancer tissues. In normal fetal and adult tissues, p-mTOR staining was observed in the intestinal crypt, intrahepatic bile ductule, pancreatic duct, distal nephron of the kidney, umbrella cell of urothelium, mesothelial cell, and choroid plexus. In cancer tissues, p-mTOR expression was higher in adenocarcinoma than in other types of cancers, in metastatic cancer than in primary cancer, and in the forefront of the infiltrating cancer cells. These results suggest that p-mTOR is implicated not only in cell proliferation but also in tubular morphogenesis in normal and cancer tissues. In addition, mTOR activation appears to be associated with cancer cell invasion and migration in solid tumors.

## 1. Introduction

Mammalian target of rapamycin (mTOR) is a member of PI3K/Akt/mTOR signaling pathway, which is involved in the regulation of numerous cellular processes including metabolism, macromolecular synthesis, growth, and survival [1]. It is also critical in the control of human embryonic stem cell (SC) growth and proliferation [2].

mTOR is a component of two multiprotein complexes, mTOR complex 1 (mTORC1) and mTORC2. In addition to mTOR, mTORC1 contains Raptor (regulatory-associated protein of mTOR), PRAS40, Deptor, and mLST8, whereas mTORC2 contains Rictor (rapamycin-insensitive companion of mTOR), mSIN1, Deptor, and mLST8 [1]. mTORC1 regulates phosphorylation of p70 S6 kinase (S6K) and eukaryotic initiation factor 4E binding protein 1 (4E-BP1), leading to protein synthesis [1]. mTORC1 is activated by Akt, while mTORC2 activates Akt by phosphorylation of Ser473 and regulates the actin cytoskeleton [1].

mTOR is deregulated in many disease conditions [1], and upregulation of mTOR is implicated in the development of cancer and the maintenance of cancer SC (CSC) [3]. Currently, numerous mTOR inhibitors (rapamycin or rapamycin analogues) are being developed and many are tried in the clinics for the cancer patients [4]. The activity of mTOR is indicated by phosphorylation of mTOR at Ser2448 (p-mTOR) [5]. p-mTOR overexpression has been reported in cancers of the breast [6], pancreas [7], liver [8], colon [9], and lung [10]; however, the overall characteristics of p-mTOR expression commonly present in these cancers have not been fully investigated. Moreover, the expression pattern of p-mTOR in human normal tissue is largely unknown.

In the present study, an immunohistochemical analysis was performed to explore the expression pattern of p-mTOR in human normal fetal and adult tissues and various carcinoma tissues. Comprehensive expression profiling of p-mTOR in human tissues would provide an insight into diverse roles of this molecule in normal and cancer tissues.

## 2. Materials and Methods

### 2.1. Tissue Samples and Tissue Microarrays

This study was approved by the Institutional Review Board of Nowon Eulji Medical Center. To examine the immunohistochemical staining for p-mTOR in human normal and cancer tissues, we used previously constructed tissue microarrays (TMAs) of normal human fetal and adult tissues [11] and urothelial carcinoma (UC) [12]. Two fetal tissues of 21 and 38 weeks of gestational age and 42 normal adult tissues from different organs and 102 UC tissues were included in TMAs [11, 12]. Additionally, 32 carcinoma tissues from the lung, liver, pancreas, stomach, colon, and kidney were obtained from tissue archives within the Department of Pathology at Nowon Eulji Medical Center, Eulji University, Seoul, Korea. These tissues were prepared with whole section from formalin-fixed, paraffin-embedded tissue blocks.

### 2.2. Immunohistochemistry

Immunohistochemical staining was performed using Dako Autostainer (DakoCytomation, Carpinteria, CA, USA). Four micron-thick tissue sections were obtained from TMA blocks and transferred onto poly-L-lysine-coated slides. After deparaffinization and rehydration, antigen retrieval was performed using citrate buffer (pH 6.0) at 121°C for 10 minutes. Endogenous peroxidase activity was blocked with 3% hydrogen peroxide for 5 minutes, and the sections were incubated with antibodies against p-mTOR (Ser2448) (Cell signaling, Danvers, MA, USA, 1 : 250). Color was developed with diaminobenzidine, and the slides were counterstained with hematoxylin. Breast carcinoma was used as a positive control for p-mTOR. Cases that omitted primary antibody served as negative control. The membranous and/or cytoplasmic expression of p-mTOR was approved as positive staining. The staining intensity with the number of positive cells was scored as follows: −, undetectable; ±, <5% positive cells; 1+, mild intensity in most cells; 2+, moderate intensity in most cells; and 3+, strong intensity in most cells.

## 3. Results

The expression profiles of p-mTOR in normal and carcinoma tissues were summarized in Tables 1 and 2, respectively.

### 3.1. Expression of p-mTOR in Normal Human Tissues

**Figure 1:**
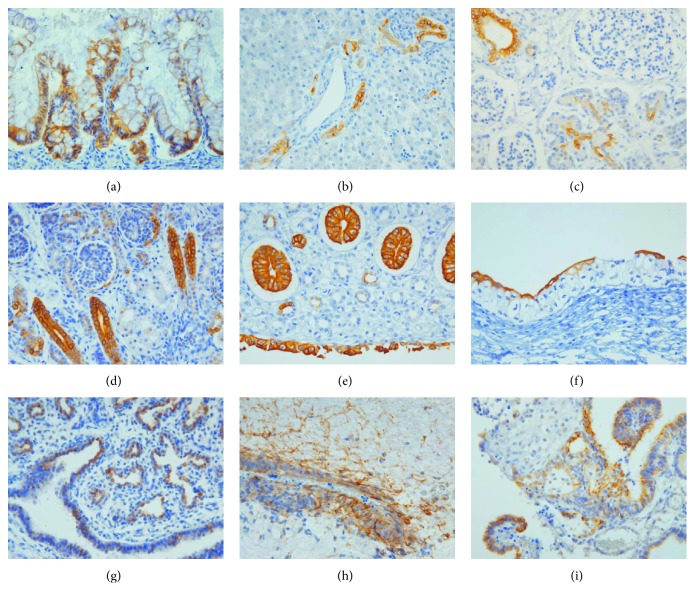
Immunoexpression of p-mTOR in human fetal tissues (400x): (a) large intestine, (b) liver, (c) pancreas, (d) kidney, (e) renal papilla, (f) urothelium, (g) lung, (h) ependyma with glial fibrils, and (i) choroid plexus of the brain.

**Figure 2:**
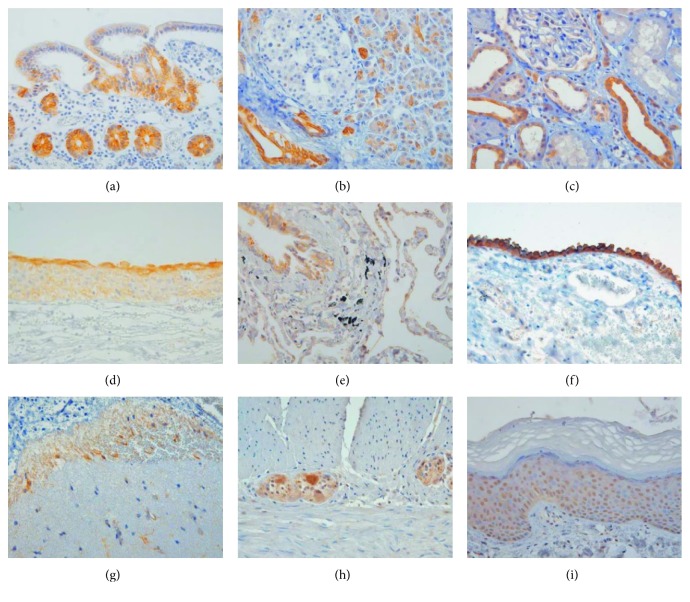
Immunoexpression of p-mTOR in human adult tissues (400x): (a) small intestine, (b) pancreas, (c) kidney, (d) urothelium, (e) lung, (f) mesothelium, (g) brain cortex, (h) myenteric plexus, and (i) skin.

p-mTOR expression pattern was similar in normal human fetal and adult tissues. The expression of p-mTOR was localized in the specific epithelial zone in internal organs, such as the intestine, stomach, liver, pancreas, kidney, and bladder. In the small and large intestine, p-mTOR showed intense membranous staining with/without cytoplasmic staining in the cells located at the crypt (Figures 1(a) and 2(a)). In the stomach, p-mTOR staining was weakly seen in the isthmic region of the antrum and corpus. In the liver, p-mTOR was only stained in bile ductules (canals of Hering) (Figure 1(b)). In the pancreas, ductal and centroacinar cells were intensely stained for p-mTOR, while islet and acinar cells were p-mTOR negative (Figures 1(c) and 2(b)). In the kidney, p-mTOR expression was occasionally found in Bowman's capsule and proximal tubule and strong in the distal tubule (Figures 1(d) and 2(c)), collecting duct, and renal papillae (Figure 1(e)). In the urinary bladder, p-mTOR expression was exclusively stained in the umbrella cells of fetal urothelium (Figure 1(f)) and extended to whole layer of the adult urothelium with still strongest staining in the umbrella cells (Figure 2(d)). In fetal lung, p-mTOR staining was strong in the pneumocytes and terminal bronchiole and rarely seen in the bronchus (Figure 1(g)), while in adult lung, p-mTOR staining was more frequently seen in bronchial epithelium than in the pneumocytes (Figure 2(e)).

The mesothelial cells covering the serosa of the gastrointestinal tract and pleura were consistently p-mTOR-positive in fetal and adult tissues (Figure 2(f)). In fetal brain, p-mTOR expression was strong in the cytoplasm of ependymal cells with their glial fibrils (Figure 1(h)), choroid plexus epithelial cells (Figure 1(i)), and pia mater, while it was faint in the nuclei of neuronal cells in the subventricular zone and cortical plate. In adult brain, p-mTOR was weakly stained in the pia mater and occasionally seen in glial cells in the cortical area (Figure 2(g)). Outside the brain, ganglion within myenteric plexus in the gastrointestinal tract showed strong cytoplasmic p-mTOR staining (Figure 2(h)).

In adult skin, p-mTOR was more intensely stained in the nucleus than the cytoplasm of squamous cells (Figure 2(i)) and sweat glands. However, the evaluation of p-mTOR staining in fetal skin was not available because fetal skin could not be obtained. In mesenchymal elements, p-mTOR was occasionally positive in the nuclei of lymphoid cells, endothelial cells, fibroblasts, and smooth muscle cells.

### 3.2. Expression of p-mTOR in Carcinoma Tissues

**Figure 3:**
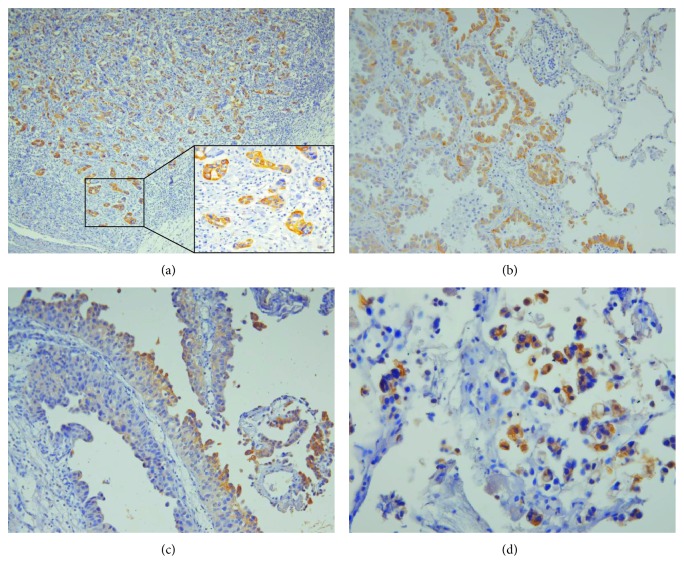
Immunoexpression of p-mTOR in carcinoma tissues. (a) Pancreatic adenocarcinoma shows stronger p-mTOR staining in the invasive front of the tumor (100x). The insert (right lower) shows higher magnification (400x). (b) In lung adenocarcinoma, p-mTOR staining is intensified at the tumor-normal interface (200x). (c) In papillary urothelial carcinoma, p-mTOR staining is stronger in the superficial layer of the tumor (200x). (d) In micropapillary variant of urothelial carcinoma, tumor cells show strong cytoplasmic and membranous p-mTOR staining (400x).

p-mTOR was expressed in most adenocarcinomas (ACs) analyzed, including those derived from the stomach, colon, pancreas, and lung. p-mTOR showed membranous and/or cytoplasmic staining in the tumor cells with variable intensity depending on the tumor phenotype. In gastric ACs, p-mTOR was heterogeneously stained in intestinal type (4/5) but negative in diffuse type containing signet ring cells (1/5). In lung ACs, p-mTOR staining intensity was higher in the well-differentiated type than in the poorly differentiated type. In colonic and pancreatic ACs, p-mTOR staining was diffuse in overall tumor cell population but intensified at the invasive front of the tumor (Figure 3(a)). Similarly, p-mTOR staining in lung AC was stronger at the interface between the tumor and normal tissues (Figure 3(b)).

In UC, p-mTOR was positive in 23/102 (23%) cases and there was no significant correlation between p-mTOR positivity and clinicopathologic parameters, such as tumor grade, stage, and lymphovascular invasion. The remarkable finding was the localization of p-mTOR staining, which was intense in the superficial layer of papillary UC (Figure 3(c)) and micropapillary variant of UC (Figure 3(d)) and in the invasive front in a subset of the muscle-invasive UC.

In the case of squamous cell carcinoma (SCC), p-mTOR showed different staining patterns depending on the tumor sites. Whereas SCC arising from the skin showed diffuse and moderate p-mTOR expression, SCC from lung showed low frequency and intensity of p-mTOR stain.

**Figure 4:**
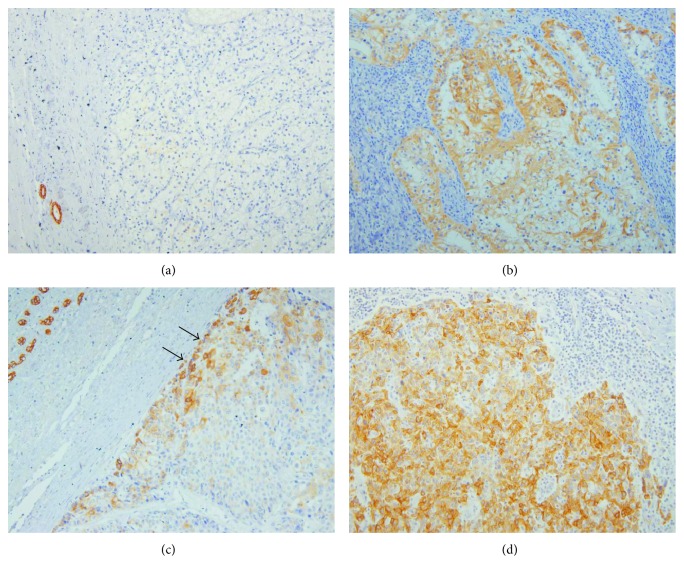
Immunoexpression of p-mTOR in primary and metastatic carcinoma tissues (200x). (a) Primary clear cell renal cell carcinoma shows p-mTOR negativity, while (b) metastatic cancer in the lymph node of the same patient shows strong p-mTOR positivity. (c) Hepatocellular carcinoma shows patchy p-mTOR staining along the periphery of the tumor in the liver (arrows) and (d) metastatic cancer in the lymph node shows diffuse p-mTOR positivity.

In the kidney, p-mTOR staining was negative or focally positive in clear cell renal cell carcinoma (RCC) (Figure 4(a)), while p-mTOR staining was strong in metastatic RCC (Figure 4(b)). Likewise, p-mTOR staining was more diffuse and stronger in metastatic hepatocellular carcinoma (HCC) than in primary HCC (Figures 4(c) and 4(d)). Additional finding in HCC was that p-mTOR staining was intensified in the periphery of the primary tumor.

## 4. Discussion

In the present study, we found a specific distribution of p-mTOR in normal and carcinoma tissues. In normal tissues, p-mTOR was selectively expressed in the intestinal crypt, bile ductules of the liver, pancreatic ductal cells, and distal nephron of the kidney, in which SCs or transit amplifying cells, also termed progenitor cells, are known to be located, although there are still debates on their location in the kidney [13–16]. The expression pattern of p-mTOR in the normal intestine, pancreas, kidney, and bladder tissues was consistent with the earlier immunohistochemical data [7, 9, 17, 18].

The intestinal crypt, a multipotent SC niche, generates new cells and TSC2/mTORC1 signaling regulates intestinal epithelial differentiation and homeostasis in a Notch-dependent manner [19]. TOR is indeed involved in intestinal epithelial morphogenesis, which is evolutionally conserved [20]. In the pancreas, candidate stem/progenitor cells are thought to reside in the pancreatic ducts, where they differentiate into multiple pancreatic lineage cells [16, 21]. Li et al. [22] reveal that adult pancreatic duct cells contribute to regeneration of the pancreas after injury, recapitulating embryonic pancreas differentiation process. Because pancreatic ductal cell differentiation is mediated by PI3K/Akt pathway [23], p-mTOR expression in the ductal cells may indicate the involvement of mTOR activation in pancreas differentiation and regeneration. In the kidney, mTOR regulates normal renal function, and dysregulation of mTOR signaling contributes to kidney diseases like diabetic nephropathy and cystic kidney disease [17, 24].

In line with strong p-mTOR staining in the renal papilla, p-mTOR was intensely stained in the umbrella cells of the urothelium, corresponding with previous results [18, 25]. The umbrella cells are known as terminally differentiated cells of the urothelium, which perform diverse functions, such as barrier, modulator of the urine, and a sensory web transmitting the information from the urinary space to the underlying nervous and muscular systems [25, 26]. In fetal brain, we found strong p-mTOR expression in the choroid plexus. The choroid plexus produce cerebrospinal fluid, which functions as a fluid cushion, and has many other functions such as the control of the neural SC migration and a communication between the brain and the rest of the body [27]. Likewise, mesothelial cells perform similar functions, such as lubricant production, the transport of fluid, leukocyte migration, and sensing and responding to external signals [28]. The expression of p-mTOR in mesothelial cells, choroid plexus, and umbrella cells of urothelium suggests a role of mTOR activation in homeostatic regulation in the internal organs.

In cancer tissues examined in this study, p-mTOR staining showed several characteristics. First, p-mTOR positivity was higher in AC than in other types of cancers, as shown in the literature [10, 18, 29]. Dobashi et al. [10] describe that in lung cancers, the frequency of p-mTOR staining is higher in AC than in SCC, and p-mTOR staining intensity in AC is stronger in the well-differentiated subtype than in the undifferentiated tumor, consistent with our findings. Likewise, Melling et al. [29] report that in the colorectal cancer, p-mTOR staining is more frequent in tubular adenocarcinomas than in other histologic subtypes (mucinous, medullary, and signet cell). In bladder cancers, p-mTOR staining was positive in a subset of invasive UC and intense in the superficial layer of papillary UC, which is in concordant with earlier data [18, 25]. We additionally found strong p-mTOR staining in micropapillary UC, which is characterized by small tight clusters of tumor cells within lacunae [30]. These results suggest that specific p-mTOR expression pattern in normal tissues is preserved in the cancer tissues and mTOR activation is possibly involved in the morphogenesis of acinar structure in both normal and cancer tissues. The mechanism of mTOR involvement in acinar structure formation is largely unknown, but there are studies showing an important role of mTOR in this process [31, 32]. mTOR modulates epithelial tubule formation in MDCK cells [31]. mTORC2 is necessary for mammary epithelial cell branching morphogenesis, survival, and motility, and mTOR directs these processes through a PKC-alpha/Rac1-dependent mechanism [32].

The second remarkable finding was that p-mTOR expression was higher in metastatic cancer than in primary cancer of the liver and kidney. Our result showing stronger p-mTOR expression in metastatic RCC and HCC than in their primary counterparts is consistent with the previous immunohistochemical data [33] and supports the rationale for clinical use of mTOR inhibitors in advanced RCC and HCC [34, 35].

The third finding of p-mTOR staining in examined cancer tissues was intense p-mTOR staining in the periphery or invasive front of the tumor, which was seen in ACs of the lung, colon, and pancreas. This peculiar p-mTOR pattern in the cancer is not widely assessed, but Wen et al. [36] show that p-mTOR staining is significantly higher in the forefront of tumor infiltrating cells of stage IIIB colon cancer and associated with increasing mortality risk of colon cancer patients. In colorectal cancer tissues, the strongest nuclear *β*-catenin accumulation is observed in dedifferentiated tumor cells at the tumor-host interface, and these cells are speculated as “mobile CSCs” [37]. The “mobile CSCs” are derived from “stationary CSCs” and acquire the traits of stemness and epithelial-mesenchymal transition (EMT) [37]. Elevated mTOR activity regulates EMT, motility, and metastasis of colorectal cancer via RhoA and Rac1 signaling pathways [38]. mTOR is a nuclear-cytoplasmic shuttling protein, and cytoplasmic p-mTOR localization corresponds with the organization of actin cytoskeleton, leading to cell migration [1, 39]. Stronger membranous and/or cytoplasmic p-mTOR staining in tumor cells at the invasive front and metastatic cancer seen in our study might indicate the involvement of p-mTOR in CSC mobility.

mTOR pathway is intricately linked with diverse signaling mediators, including glycogen synthase kinase 3*β* (GSK3*β*) [40]. GSK3*β* is a multifunctional kinase and member of Wnt/*β*-catenin pathway, which is implicated in normal tissue development and tumor initiation as well as SC fate control [41, 42]. GSK3*β* and mTOR are downstream molecules of Akt and at the same time effector molecules of each other [40, 43]. Mechanical regulation of GSK3*β* is dependent on mTORC2 activation and Akt phosphorylation at Ser-473 in mesenchymal SCs [43]. In dairy cow mammary epithelial cells, pS9GSK3*β* enhances the mTOR/S6K1 pathway leading to cell proliferation and milk synthesis, but GSK3*β* represses this pathway [40]. Previously, we have reported the specific distribution of pS9GSK3*β* in normal tissues, such as intestinal crypt, bile ductules of the liver, pancreatic ductal and centroacinar cells, distal nephron of the kidney, urothelium, lung, and mesothelial cells [11], which is strikingly in accordance with p-mTOR expression shown in this study. We also found similar expression of cytokeratin7 (CK7), cytoskeletal intermediate filament, in the liver, pancreas, kidney, and mesothelial cells [11]. Taken together, these results suggest that p-mTOR and pS9GSK3*β* positively correspond to the control of cell growth and epithelial differentiation, probably associated with CK7 in a subset of normal tissues.

## 5. Conclusion

The specific distribution of p-mTOR indicates that this molecule is involved not only in growth control but also in tubular structuring of normal and cancer tissues. Moreover, mTOR activation appears to be associated with cancer cell invasion and metastasis in solid tumors. In addition, different expression status of p-mTOR depending on different histologic types of cancer suggests that p-mTOR may be an immunohistochemical marker for guiding the judicious application of p-mTOR inhibitors in an individual cancer patient, although further study on whether the treatment response is different according to different p-mTOR patterns of cancer is required.

## Figures and Tables

**Table 1:** Expression of p-mTOR in normal human fetal and adult tissues.

Organ	Cell type	p-mTOR	
		Fetus	Adult
Stomach	Foveolar epithelium	+	+
	Glands	±	±
Bowel	Crypt	+++	+++
	Mesothelial cell	+++	+++
	Myenteric plexus (ganglion)	+	++
	Myenteric plexus (myelin sheath)	+	+

Liver	Hepatocyte	−	−
	Bile duct	+++	+++
Pancreas	Islet	−	−
	Acinar	−	−
	Centroacinar	+++	+++
	Duct	+++	+++

Kidney	Bowman's capsule	±	±
	Proximal tubule	±	±
	Distal tubule	+++	+++
	Collecting duct	+++	+++
Bladder	Urothelium (umbrella cell)	+++	+++
	(basal to intermediate cell)	−	+

Lung	Bronchial epithelium	±	++
	Pneumocyte	+++	±

Brain	Neuron	+(n^∗^)	±
	Neuroglia (nonependymal cell)	±	±
	Ependymal cell	+++	NA
	Choroid plexus	++	++

Skin	Squamous epithelium	NA	+(n^∗^)
	Sweat gland	NA	+

−: undetectable; ±: <5% positive cells; +: mild intensity in most cells; ++: moderate intensity in most cells; +++: strong intensity in most cells; (n^∗^): nuclear staining; NA: not available.

**Table 2:** Expression of p-mTOR in carcinoma tissues: a survey of 134 cases.

Organ	Carcinomas	Total cases	p-mTOR (+) cases	Intensity
Stomach	Adenocarcinoma	5	4	++
Colon	Adenocarcinoma	4	4	+++
Pancreas	Adenocarcinoma	2	2	+++
Lung	Adenocarcinoma	4	4	++
	Squamous cell carcinoma	3	1	+
Liver	Hepatocellular carcinoma	5	2	++
Kidney	Renal cell carcinoma	3	1	±
Bladder	Urothelial carcinoma	102	23	++
Skin	Squamous cell carcinoma	2	2	++
Lymph node	Metastatic hepatocellular carcinoma	2	2	+++
	Metastatic renal cell carcinoma	2	2	+++

±: <5% positive cells; +: mild intensity in most cells; ++: moderate intensity in most cells; +++: strong intensity in most cells.
